# Enhancing Neurology Clerkship Training With Problem-Based Learning and the Mini-Clinical Evaluation Exercise (Mini-CEX)

**DOI:** 10.7759/cureus.94481

**Published:** 2025-10-13

**Authors:** Yingfang She

**Affiliations:** 1 Neurology Medicine Center, The Seventh Affiliated Hospital of Sun Yat-sen University, Shenzhen, CHN

**Keywords:** clinical clerkship, competency-based education, medical education, mini-cex, neurology, problem-based learning

## Abstract

Background: Neurology clerkships are a critical stage in medical education, where students must connect theoretical knowledge with clinical practice. Traditional methods often fall short in developing clinical reasoning, procedural skills, and professional competencies.

Objective: This review examines the potential of combining problem-based learning (PBL) with the mini-Clinical Evaluation Exercise (mini-CEX) to strengthen neurology clerkship training.

Methods: We conducted a narrative review of recent studies on PBL and mini-CEX in neurology and related fields, including evidence from systematic reviews, randomized trials, and observational research.

Results: PBL enhances knowledge, self-directed learning, and engagement. Mini-CEX provides structured, real-time assessment with immediate feedback. When used together, they show synergistic benefits in other specialties, improving exam performance, clinical reasoning, communication, and learner satisfaction. Key challenges include faculty workload, rating variability, and limited neurology-specific evidence.

Conclusions: Integrating PBL with mini-CEX offers a competency-based approach to neurology clerkships. Early evidence supports its feasibility and educational value, but further undergraduate-focused studies are needed. Successful implementation will depend on careful design, faculty training, and institutional support.

## Introduction and background

Neurology clerkships are widely recognized as pivotal for consolidating clinical reasoning and bedside examination skills. The term “neurophobia” was first described by Jozefowicz in 1994, referring to low confidence and perceived difficulty in neurology, and continues to undermine learners’ engagement and performance [1]. Recent evidence confirms that neurophobia remains widespread among medical students and junior doctors worldwide. A systematic review and meta-analysis of 24 studies involving 10,395 participants across 30 countries estimated an overall pooled prevalence of 46% (95% CI, 35-57%), with regional variation significantly influencing prevalence rates [2].

Concurrently, undergraduate programs are shifting toward competency-based medical education (CBME), which emphasizes observable performance, longitudinal feedback, and alignment of assessment with real clinical work. This shift requires curricula to pair active, learner-centered methods with authentic workplace-based assessments that generate actionable feedback [3].

Problem-based learning (PBL) is a well-established, student-centered approach that can strengthen clinical competence, satisfaction, and higher-order thinking. Meta-analytic evidence from the past few years shows PBL outperforming lecture-based learning for clinical competence in several disciplines [4-6], and recent syntheses indicate PBL can meaningfully enhance medical students’ critical thinking - an essential precursor to clinical reasoning in neurology [7]. Neurology clerkships are particularly suited to this approach, as they require complex reasoning for lesion localization and differential diagnosis, combined with direct observation of examination and communication skills.

Complementing PBL, the mini-Clinical Evaluation Exercise (mini-CEX) provides structured, direct observation with immediate feedback across core competencies in authentic encounters. Contemporary randomized and systematic studies report the mini-CEX to be feasible, acceptable, and educationally valuable for undergraduate learners, with neurology-focused work at the postgraduate level also demonstrating improved skills and positive stakeholder perceptions [8,9].

Taken together, these trends support integrating PBL (to activate and organize clinical reasoning around real problems) with the mini-CEX (to observe performance, calibrate expectations, and deliver targeted feedback at the bedside). Emerging evidence from hybrid, assessment-rich models suggests such combinations can amplify gains in clinical judgment and overall competence - precisely the outcomes targeted in a neurology clerkship [10].

## Review

Methods

To inform this review, we conducted a structured literature search of PubMed and Web of Science covering the period from January 2015 to June 2025. Search terms included combinations of “Problem-Based Learning,” “mini-Clinical Evaluation Exercise,” “neurology clerkship,” and “clinical education.” Eligible studies were English-language publications that evaluated educational applications of PBL or mini-CEX in undergraduate or postgraduate medical training. We included systematic reviews, randomized controlled trials, and observational studies that reported educational outcomes such as knowledge acquisition, clinical reasoning, skill performance, communication, or learner satisfaction. Exclusion criteria were commentaries, conference abstracts, and studies not directly addressing educational outcomes. Reference lists of key reviews were also screened for additional relevant publications. The included evidence was synthesized narratively to highlight educational impact, implementation strategies, and reported challenges.

Theoretical foundations of PBL and the mini-CEX

PBL is a student-centered pedagogy in which small groups of learners tackle authentic clinical problems under facilitator guidance [11,12]. It is grounded in constructivist and collaborative learning theories: students build new knowledge on prior experience, learn actively through cases, and develop critical thinking by solving real-world problems [13]. In practice, PBL sessions are discussion-based and student-driven, fostering autonomy, reflection, and teamwork [14]. Evidence indicates that PBL organizes learning around clinical cases and enhances motivation and deep understanding [15]. In neurology, PBL (alone or combined with case-based learning) has been shown to raise exam scores, self-learning skills, and overall satisfaction compared to lectures [16].

The mini-CEX, originally developed by Norcini, is a validated tool for workplace-based assessment of clinical competencies across seven domains [17]. A typical encounter lasts 15-20 minutes, during which a supervisor observes a trainee with a real patient and provides immediate feedback [18]. Mini-CEX targets the “does” level of Miller’s pyramid [19] and emphasizes feedback and reflection in authentic contexts. In neurology clerkships, it has been used to strengthen both cognitive and interpersonal skills, addressing gaps left by traditional exams [18,20]. To clarify its scope, Table 1 summarizes the seven domains of the mini-CEX and their relevance to neurology clerkships.

**Table 1:** Domains of the Mini-Clinical Evaluation Exercise (mini-CEX) and Relevance to Neurology Clerkships

Domain	Description	Relevance in Neurology Clerkships
History-taking	Ability to obtain accurate, comprehensive, and focused clinical history	Critical for neurological localization (e.g., onset, progression, risk factors for stroke, seizure)
Physical examination	Competence in performing systematic and targeted examinations	Essential for neurological exam (cranial nerves, motor/sensory systems, coordination, reflexes)
Clinical judgment	Formulating differential diagnoses, diagnostic reasoning, and management plans	Core to addressing neurophobia by reinforcing reasoning in lesion localization and decision-making
Communication skills	Interaction with patients and families, explaining diagnoses and plans	Important for discussing complex neurological conditions, informed consent, and breaking bad news
Professionalism	Ethical behavior, empathy, respect, and responsibility	Builds trust with patients/families in sensitive neurology settings (e.g., dementia, epilepsy)
Organization/Efficiency	Managing time, prioritizing tasks, structuring encounters	Necessary in acute neurology scenarios (stroke code, emergency seizure management)
Overall clinical competence	Global rating that integrates all the above domains	Provides a holistic judgment of student readiness for neurology practice

PBL in neurology clerkships

Clinical internships mark the transition from classroom knowledge to independent clinical practice and demand competence in reasoning, communication, and procedural skills. PBL has been successfully applied in neurology teaching [12,21]. Instead of focusing on isolated outcomes from individual studies, the collective evidence suggests that PBL enhances theoretical knowledge, clinical reasoning, and learner motivation [16,21,22]. Importantly, PBL also mitigates the widely recognized challenge of neurophobia by engaging students in active problem-solving: learners report greater enthusiasm and confidence in diagnosing neurological problems [16,23]. More recent innovations, such as combining PBL with online small private courses, further improve engagement and align with modern educational needs [24]. Together, these findings support PBL as a promising approach to reduce neurophobia and strengthen neurology clerkship training.

Mini-CEX in neurology clerkships

Mini-CEX has been adopted internationally as a formative tool in undergraduate and postgraduate education [25-27], with relatively consistent application across diverse cultural contexts [28]. In neurology, repeated mini-CEX encounters have improved residents’ clinical and humanistic skills, with positive responses from both trainees and faculty [20]. Across specialties, a meta-analysis confirms that mini-CEX enhances learner performance, though outcomes are moderated by implementation quality [19]. One limitation is subjectivity: ratings may be influenced by rater or context, underscoring the need for multiple encounters and trained assessors [18]. In neurology settings, however, these challenges appear manageable, and students generally respond well to structured feedback [27].

Integration of PBL and mini-CEX in clinical teaching

**Figure 1:**
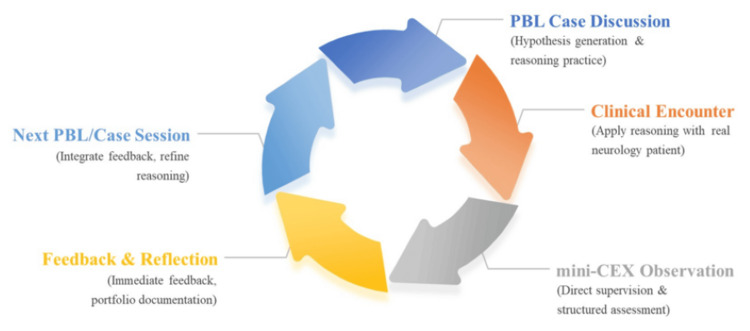
Conceptual model for integrating problem-based learning (PBL) with the mini-Clinical Evaluation Exercise (mini-CEX) in neurology clerkships The cycle begins with a PBL case discussion, where students generate hypotheses and practice diagnostic reasoning. Learners then apply this reasoning in real clinical encounters, which are directly observed and assessed through the mini-CEX. Immediate feedback and reflection follow, with documentation in portfolios or e-portfolios. This feedback is reintegrated into subsequent PBL sessions, creating a continuous loop that links classroom reasoning, bedside performance, and reflective learning.

While PBL fosters reasoning, it may create fragmented knowledge if not connected to real patient encounters [12,29,30]. Mini-CEX complements this by providing structured observation and feedback in authentic settings. Although no undergraduate neurology study has directly combined both methods, evidence from nephrology and pediatrics shows that integration improves knowledge, case analysis, communication, and overall competence [31,32]. We therefore propose a conceptual model where PBL case discussions are scheduled upstream of clinical shifts, enabling learners to apply hypotheses in real encounters observed via mini-CEX, followed by structured feedback and portfolio reflection. This sequence (Figure 1) represents a cycle linking classroom reasoning, bedside performance, and reflective learning. Although current evidence is largely indirect and drawn from other specialties, this model offers a practical framework for neurology clerkships aligned with competency-based medical education principles.

Practical implementation strategies

A pragmatic pathway for integrating PBL with the mini-CEX in neurology clerkships involves six interlocking components: curriculum design, scheduling, assessment structure, feedback culture, faculty development, and digital infrastructure. In practice, programs align PBL cases with common neurological syndromes, distribute multiple short observations across different supervisors, and embed mini-CEX ratings within a broader portfolio-based assessment framework. Implementation quality-particularly timely, behavior-specific feedback and trained raters-emerges as a key determinant of educational impact [2,11,33-35]. Faculty development and lightweight digital platforms further support feasibility, allowing coordination of PBL case materials, scheduling, documentation, and analytics [36,37].

Barriers remain, however, including limited faculty time, variability in assessment quality, and logistical challenges of aligning cases with clinical encounters. These can be addressed through distributed observation schedules, structured rater training, and portfolio integration. Table 2 summarizes common barriers and corresponding strategies for effective implementation.

**Table 2:** Barriers and strategies for integrating problem-based learning (PBL) with the mini-Clinical Evaluation Exercise (mini-CEX) in neurology clerkships This table summarizes common barriers encountered when combining PBL and mini-CEX—such as faculty time constraints, variability in rater judgments, and scheduling difficulties—and provides practical strategies to address each barrier. Supporting references are included to highlight the evidence base for these solutions. The table is intended as a quick-reference framework to complement the description in the Practical Implementation Strategies section.

Barrier	Strategy/Implementation	Supporting Evidence
Limited faculty time	Use brief (10–15 min) mini-CEX encounters distributed across supervisors	[11,33]
Variability in ratings	Provide rater training, shared descriptors, and entrustment scales	[2,21]
Fragmented knowledge from PBL	Align PBL cases with real patient opportunities (“case-to-clinic” pairing)	[25,32]
Feedback inconsistency	Emphasize immediate, behavior-anchored feedback with portfolio documentation	[22,35]
Logistical/scheduling challenges	Employ electronic platforms for scheduling, case-linking, and feedback capture	[36,37]

Benefits for neurology training

In brief, the integrated PBL-mini-CEX model supports four complementary benefits for neurology clerkships (Figure 1, Table 1).

Reasoning Transfer

PBL structures hypothesis generation while mini-CEX requires applying that reasoning with real patients, reinforcing “learning by doing” and critical thinking [16,20].

Competence Gains

Synthesized evidence shows improvements in knowledge and skills, with performance gains when observation is embedded within repeated, brief encounters [19,31].

Learner Engagement

Active, case-driven learning and direct observation are associated with higher learner enthusiasm and satisfaction across neurology-adjacent contexts [20,22].

Feedback and CBME Alignment

Immediate, behavior-specific feedback during mini-CEX strengthens bedside performance and maps onto the core competency domains (history, exam, judgment, communication, professionalism) summarized in Table 1; educational impact increases with implementation quality [19].

Challenges and limitations

Several practical barriers must be acknowledged, as seen below.

Faculty and Time Resources

PBL and mini-CEX are labor-intensive. Mini-CEX encounters typically take 10-20 minutes including feedback [20]. Allocating this time during busy ward schedules can be challenging. PBL groups require multiple facilitators and planning. Departments must commit faculty time and may need to adjust patient loads. Mitigation: distribute brief observations across multiple supervisors; schedule “micro-mini-CEX” during routine workflow; involve near-peer teaching assistants (senior students/residents) for PBL facilitation under faculty oversight; and use digital tools for scheduling/forms to reduce coordination overhead [34,35].

Assessment Variability

As Rogausch et al. note, mini-CEX ratings can suffer from subjectivity and context effects. Ensuring reliability demands multiple assessments and possibly standard-setting for scores [18]. Inconsistent feedback quality can limit benefit. Similarly, poorly structured PBL can degenerate into unproductive discussions. Faculty training is key to mitigate these issues. Mitigation: implement brief rater training and shared descriptors/entrustment anchors; diversify assessors and settings to average out context effects; embed periodic norming sessions; and aggregate evidence in portfolios to improve decision reliability [33,38].

Integration Difficulty

Aligning PBL session topics with actual patient opportunities can be logistically tricky. Neurology clerkships cover acute and outpatient care; some essential cases (e.g., rare neuromuscular diseases) might not present during a rotation, limiting mini-CEX variety. For mitigation, pair each PBL case with pre-identified ward/clinic slots; supplement scarce presentations with simulation/standardized patients and brief skills stations; and maintain a running “case map” to track exposure gaps and redirect learners accordingly [22,31,32].

Learner Adjustment

Students unfamiliar with PBL may initially struggle with self-directed learning or feel uncomfortable being evaluated in real time. They may need orientation to the PBL process and reassurance about the formative nature of mini-CEX. Mitigation involves providing upfront orientation and expectations, feedback-literacy briefing, and low-stakes early observations before higher-stakes encounters [19].

Limited Evidence Base

Few studies have examined PBL + mini-CEX in undergraduate neurology. Most evidence comes from other specialties, such as surgery [4] and pediatrics [32], where integration improved knowledge, reasoning, and learner satisfaction. These results suggest broad applicability, but neurology’s unique demands still require specialty-specific trials to confirm transferability.

Equity Considerations

To ensure fair access to cases and observation opportunities, programs can rotate learners through standardized case lists, randomize or balance assessor assignments, and monitor portfolios/e-portfolios for exposure parity (e.g., number and type of encounters, feedback frequency). Equity guidance in assessment further supports transparent criteria and routine bias checks [39], while digital dashboards can flag disparities early for corrective action [34,35].

Future directions and practical considerations

Stronger Evidence in Undergraduate Neurology

Most integration studies currently arise from other specialties or postgraduate contexts. Multi-site trials and mixed-methods studies in undergraduate neurology would clarify effects on shelf performance, workplace skills, and neurophobia, and would help identify mechanisms (e.g., feedback quality, assessment frequency) that drive benefit. Recent meta-analytic data on neurophobia underscore the relevance of such outcomes [2].

Validity and Decision-Making Within Programmatic Assessment

Future work could examine how mini-CEX data, PBL artifacts, and simulation results contribute to high-stakes progression decisions under a programmatic approach, including generalizability, fairness, and contribution of narrative evidence. Contemporary evaluations outline conditions that enable successful programmatic assessment and may guide governance and data synthesis [36].

Integration With Entrustable Professional Activities (EPAs)

Mapping neurology clerkship activities to EPAs - for example, focused neurologic assessment or initial management of a patient with altered mental status - may improve coherence between observed tasks and progression benchmarks. Resources from national initiatives (e.g., the AAMC Core EPAs) and recent undergraduate EPA scholarship provide templates for such alignment [37].

Technology-Enabled Implementation

Digital tools for scheduling, capturing observations, and curating portfolios are increasingly described, with mobile workplace-based assessment (WBA) platforms and e-portfolios supporting timely feedback and longitudinal review [40]. Simulation, augmented reality (AR)/virtual reality (VR) cases, and scenario-based stations represent pragmatic complements when clinical opportunities are sparse.

Faculty and Learner Development

Sustainable models tend to pair assessor development (e.g., frame-of-reference training, feedback coaching) with student preparation for receiving and using feedback. The literature on rater training and feedback literacy suggests that such capacity-building contributes meaningfully to data quality and educational impact [41,42].

Feasibility, Cost, and Scalability

Reports from emergency, primary care, and inpatient settings indicate that short, distributed observations are feasible within service constraints, though attention to workload and scheduling remains important [43]. Cost-effectiveness and scalability analyses represent additional targets for future research as programs expand integrated PBL-mini-CEX models.

Summary of Priorities

Conduct prospective, neurology-specific undergraduate trials with predefined outcomes and follow-up; evaluate long-term outcomes such as skill retention, transfer to practice, and patient-relevant measures. Ensure equity and governance in assessment, supported by cost-effectiveness and scalability analyses [39,44,45]. Use implementation science approaches - such as standardized rater training and e-portfolio analytics - to support programmatic assessment.

## Conclusions

Integrating problem-based learning with the mini-Clinical Evaluation Exercise aligns neurology clerkships with competency-based medical education by linking classroom reasoning to supervised bedside performance. Current evidence indicates feasibility and gains in reasoning, communication, and examination skills - especially when PBL precedes observed encounters and multiple brief mini-CEX observations are curated in portfolios. However, neurology-specific trials remain scarce, and long-term outcomes are seldom reported. Moving forward, neurology educators are encouraged to pilot and rigorously evaluate integrated PBL-mini-CEX models, thereby contributing specialty-specific evidence and advancing competency-based training.
